# Swimming World Championships: Association between Success at the Junior and Senior Level for British Swimmers

**DOI:** 10.3390/ijerph18031237

**Published:** 2021-01-30

**Authors:** Inmaculada Yustres, Jesús Santos del Cerro, Stelios Psycharakis, Fernando González-Mohíno, José María González-Ravé

**Affiliations:** 1 Facultad de Ciencias de la Salud, Universidad Francisco de Vitoria, Ctra. Pozuelo-Majadahonda Km 1800, Pozuelo de Alarcón, 28223 Madrid, Spain; inmaculada.yustres@ufv.es; 2 Department of Economics and Business Statistics, University of Castilla la Mancha, San Pedro Mártir, 45071 Toledo, Spain; 3 Department of Sport, Physical Education and Health Sciences, University of Edinburgh, Edinburgh EH8 8AQ, UK; Stelios.Psycharakis@ed.ac.uk; 4 Department of Physical Activity and Sport Sciences, University of Castilla-La Mancha, Avenida Carlos III, 45071 Toledo, Spain; fgonzalezmohino@nebrija.es (F.G.-M.); josemaria.gonzalez@uclm.es (J.M.G.-R.); 5 Facultad de Educación, Universidad de Nebrija, 28240 Madrid, Spain

**Keywords:** swimming, talent, early specialization

## Abstract

The present study examines the association between the success at junior and senior level for British swimmers in World Championships (WCs). It also explores the relationships between swimming performance and the swimmers’ gender, swim stroke, distance, status (finalist, semifinalist and heats) and swimming category. Data were collected for swimmers participating in junior and/or senior World Championships (29,000 entries: 5585 swimmers) from 2006–2017. The final filtered database included only swimmers from the United Kingdom (836 entries: 141 swimmers). A descriptive analysis was made to characterize the swimmers who reached elite status in the senior category. A lineal regression model was run by gender to predict the influence of category, swim stroke, and distance in the results reached in the senior category. The results showed that the ratio of conversion from junior to senior was quite low. Females who participated in both junior and senior WCs were likely to reach top positions in the senior category. Overall, few British swimmers participated in a junior category before the senior level, but female swimmers participating in both junior and senior WCs were likely to reach top positions in the senior category.

## 1. Introduction

Coaches, clubs, federations, and governing bodies involved in competitive sports seek to maximize performance in athletes. In an effort to assess how a country can manage future success in international sporting competitions such a swimming World Championships, we here evaluate the interaction between early success in the junior category and subsequent success in the senior category, exploring the critical factors that affect this future success in international sporting competitions. In this sense, researchers have been interested in the variables that may influence performance in sports. Performance during the early stages of an athlete’s career has attracted increasing attention [1,2,3,4,5,6,7]. Several studies investigating this aspect have resulted in different opinions on the development of elite athletes across a variety of sports [8,9]. Gulbin et al. [8] showed the rate of conversion from the junior to senior level to be less than 7% among 256 elite athletes in 27 different sports, while Barreiros et al. [3] found that just a third of international junior athletes reappeared at the international senior level in different sports. Similarly, in swimming, the rate of conversions from junior to senior for elite swimmers competing in international events has also been found to be low. Yustres et al. [7] reported that just 17% of World Championship (WC) swimming finalists had previously participated in junior WC.

Some prospective studies have shown that many successful young athletes do not attain the same level of success when they reach adulthood. Moreover, those who maintain the same level are shown to follow performance progression trajectories that are generally nonlinear and that poorly predict complex ascending and descending career oscillations [9]. Costa et al. [9], tracking childhood to adulthood data on swimmers’ performances, found a constantly changing trajectory. In this sense, it is important to examine why an elite sport system in a given country is successful in specific sports and to explore the relationship between elite sport policy systems related to talent identification programs and success in international competitions.

Nevertheless, due to the increasing competition between nations for medals at major international events such as the WCs and Olympic Games [10], many national sporting organizations have invested their available resources more effectively by identifying talented athletes well in advance [11,12]. Talent identification programs aim to identify athletes with high potential for success in senior elite sports [13]. As a result of such programs, early specialization usually leads to heavier loads of training during the early stages of development of swimmers. This trend towards early specialization generally leads to a greater demand for a commitment by young athletes, creating much more physical, psychological, and social pressure [6]. The objective of early specialization is to produce and prepare world-class athletes in the shortest possible time, regardless of any negative consequences to their health and development.

Within the United Kingdom (UK), British Swimming (https://www.britishswimming.org), the national governing body for swimming, is implementing new long-term training programs with a focus on support resources and services for individual athletes and teams as well as on how these services can been delivered. This has to do with “medal conversion,” or going from talented performer to podium placement [14]. Pollock et al. [15] evaluated 18 elite British swimmers of different event specialties over a season. The individual training programs were documented by surveying the coaches, focusing more on the exercises performed, the content of training, and terminology habitually used by coaches than on giving a clue about the policymakers on the early success in junior category and subsequent success in senior category of British elite swimming. 

Yustres et al. [16], analyzing the performance-progression model of the European countries that participated in the WCs from 2006 to 2017, concluded that European countries with swimmers achieving optimal performance in the Junior WCs had a better chance of reaching the top 5 positions in the medal ranking at the Senior WCs. The present research investigates one of the best European countries in recent competition, i.e., the UK.

The UK scored the highest number of medals won in Europe during the last Swimming WC (Budapest, October 2018), European Championship (Glasgow, December 2019), and Olympic Games (Rio de Janeiro, August 2016). Thus, benefits can result from further exploring these achievements and finding any possible reasons and emerging patterns that lead to winning medals in both junior and senior swimming categories. Such analyses could be useful in identifying general success patterns, improving talent identification, and developing specific strategies for swimmers’ improvements.

One limitation of existing research is that only two studies available have examined the influence of performance in junior swimmers from all the WC competitions held, including the same swimmers’ results in elite senior categories, using a multivariate approach [7,17]. The study of Yustres et al. [7] focused on swimmers who reached the finals in WCs while the study of Yustres et al. [17] analyzed all the participants in WCs regardless of whether they reached the finals or not.

Yustres et al. [18] found that better performance at the junior WC meant higher standards in the senior WC to be more likely in all swimmers participating in this championship from 2006 to 2017. Moreover, an optimal annual performance progression from junior WC positively affected the chances of success at the senior WC.

Therefore, given the scant evidence linking early success in the junior category to subsequent success in the senior category in specific and successful national participants such as the UK, the aim of the present study was to provide a swimming-specific approach to examining the links between junior and senior success of British elite swimmers. This entails exploring the effects of gender, swim stroke, distance, status (finalist, semifinalist and heats), and category on swimmers’ performances. 

## 2. Materials and Methods

### 2.1. Participants and Measures

To assess the relationship between participation in junior WCs and success in senior WCs, we conducted an observational retrospective study, using historical data from the FINA official results websites of the 2007, 2009, 2011, 2013, 2015 and 2016 WCs and 2006, 2008, 2011, 2013, 2015 and 2017 junior WCs. The University Ethical Committee approved this study. Informed consent from participants was unnecessary because the data were publicly available. The data (29,928 entries related to 5992 swimmers) were obtained from the International junior Swimming Categories (http://www.fina.org/results). 

### 2.2. Design and Procedures

Raw data (29,928) were divided first into three categories: Category 1 (C1), swimmers who participated in senior WCs without previous participation in junior WCs (N = 3371); Category 2 (C2), swimmers who participated only in junior WCs (N = 1916); Category 3 (C3), swimmers who participated in senior WCs with previous participation in junior WCs (N = 705). Each entry included the following variables: name, race time, position, status (highest finishing position: final (3), semifinal (2), heats (1), country, gender (1 male, 2 females), distance, swim stroke, age, and year of competition. The distances analyzed were 50, 100, 200, 400, 800, 1500 freestyle (swim stroke 1); 50, 100 and 200 backstrokes (swim stroke 2)/breaststroke (swim stroke 3)/butterfly (swim stroke 4); and 200 and 400 individual medley (swim stroke 5). It bears mentioning that the first junior WCs took place in 2006. The data were therefore selected according to swimmers’ age to include only those who could have participated in the junior category due to their year of birth.

In this study, the final filtered database included only British swimmers who participated in junior and/or senior WCs (830 entries: 141 swimmers). Of these swimmers, 77 were from C1, 53 from C2, and 11 from C3.

### 2.3. Statistical Analysis

The race times were standardized by means of Z-time scores to compare swimmers’ times without any effects from gender, swim stroke, or distance. The Z-time scores were calculated by using the annual best performance of each swimmer, as follows:

$$Z_{ij}=\frac{X_{ij}-{\bar{X}}_{i}}{\sigma_{i}}\;$$

where *j* = individual, *i* = group by gender, swim stroke, and distance.

The descriptive statistics (mean, standard deviation, and frequencies) were calculated for all variables. To estimate the effect of category and style in the results reached in senior category, we applied a linear regression model. This model indicates the importance of some predictors (independent variables such as category or swim stroke) on the dependent variable (Z-time) when accounting for swimmers that participated in both junior and senior WCs [19]. The Durbin–Watson test was used to check for collinearity effects. The models were run for each gender without problems of heteroscedasticity in residuals or multicollinearity among regressors [19]. The following multiple linear regression model was used:

Z-time = β0 + β1 • Cat + β2 • St1 + β3 • St2 + β4 • St3 + β5 • St4 + β6 • St5 + εi

where: Cat = category (dummy variable with 0 for swimmers competing in junior categories and 1 for swimmers competing only in senior categories); St = Swim stroke defined as a dummy variable with 5 categories, as explained above (Swim stroke 1,2,3,4,5); β0 is the intercept of the regression model; βx are the effects of the regressors; and εi is the disturbance term. 

The effect size (ES) was calculated for a given R^2^ using Cohen’s f2. The ES interpretation was based on the following range values: 0.02 = small effect, 0.15 = medium effect, 0.35 = large effect (Cohen, 1984). All the statistical analyses were made using Excel spreadsheets and the SPSS 24.0 (IBM Corp., Armonk, NY, USA). The level of significance was set at *p* ≤ 0.05.

## 3. Results

### 3.1. British Swimmers in Junior and Senior WCs

Of the 5585 swimmers participating in WCs, 141 were British (70 males and 71 females). Of these, 81 swimmers (57.44%) competed in WCs (C1) and 60 (42.55%) in junior WCs (C2), with just 11 of those C1 and C2 swimmers participating in both categories (C3). Therefore, just 18.33% of British junior swimmers participated later in senior WCs.

As we sought to examine the effect of early success of British elite swimmers, we focused our further analysis on the 11 swimmers from the C3 category. Eight of those swimmers reached the finals with maximum status as juniors and/or seniors, two reached the semifinal, and one participated in the heats. Of the eight finalists, two reached the finals only in junior WCs, one only in senior WCs, and five in both junior and senior (J&S) WCs. When the semifinals were used as the maximum status, both swimmers participated just in junior WCs. Besides, swimmers whose maximum status corresponded to the heats participated in both J&S WCs.

### 3.2. Characterization by Distance and Swim Stroke

Descriptive data for British swimmers are presented in Table 1, expressed as a percentage of total swimmers in each stroke, distance, those who reached the finals, and percentage of females.

No swimmers were registered for 800 m and 1500 m events.

### 3.3. Regression Model

The results of the linear regression enabled us to estimate the effect of category, gender, and style in the results reached for the senior category by male and female swimmers (see Table 2). The regression models for both sexes of swimmers were statistically significant (F = 17.693; *p* < 0.001; R^2^ = 0.188; ES = 0.23; and F = 23.896; *p* < 0.001; R^2^ = 0.213; ES = 0.27, respectively) and showed that style was a significant variable (*p<* 0.05) in both cases, except for swim stroke 1 (variable excluded from both models as it did not meet the collinearity assumptions) and swim stroke 5 (*p* = 0.068 and *p* = 0.584 for males and females, respectively). In addition, the regression model for female swimmers showed a significant effect of category variable (*p* = 0.009; B = 0.199), suggesting that British females competing in the junior finals were more likely than males to compete also in the senior finals (see Table 2).

## 4. Discussion

The primary aim of the present study was to examine the link between junior and senior success at the elite level for British swimmers. The results revealed that the majority of British swimmers competed at the WC level in just one category (i.e., either junior or senior) during their career, making the ratio of conversion from junior to senior elite level quite low. Swimmers who reached the final in the junior category were more likely to compete also as seniors, compared to those who reached the semifinal or heats as juniors. Moreover, swimmers participating in both categories were likely to reach the finals at maximum status during their careers.

The low rate of conversion from junior to senior level in British swimmers in the present study (18.33%) is consistent with the results of Yustres et al. [7], who analyzed finalists from all the countries participating in WCs (conversion rate of 17%). The results from a different study for the World Junior Athletics Championships (WJC) showed that about two-thirds of the winners and medalists showed no major success as seniors [20]. These results agree with Gulbin et al. [8], who also found that the developmental linearity evident from junior to senior was quite rare (less than 7%) across 256 elite athletes in 27 different sports. Some authors have speculated that performance at the youth level is relatively independent of long-term potential due to the highly complex and nonlinear nature of athlete development [21,22,23]. These speculations roughly coincide with our results in which just 18.33% of junior swimmers reached finally significant performance in the senior category. 

Nevertheless, our analysis revealed that most of swimmers who participated in both categories reached a WC final during their careers. Moreover, swimmers who reached the finals as juniors were more likely to participate later as seniors. Consistent with this, West et al. [24] suggested that swimmers’ early success does become a significant factor in achieving better results at the senior level. A similar study reported that athletes who won medals or made the finals at the WJC in athletics, and who stayed in the sport, were more likely to go on to be successful athletes at the senior global level, compared to those who did not make the finals at the WJC [25].

Despite the above, the conversion ratio from junior to senior is still quite low. Success itself is one factor, but multiple other factors may affect a swimmer’s career at the personal and sporting support level. Thus, British swimming may benefit from exploring in further depth the possible factors contributing to the success of the British swimmers who reached the elite level as seniors, but also the reasons for those who did not make it to that level. This would boost the chances of achieving a higher conversion ratio. 

In terms of event distance, in line with the study of Yustres et al. [7], our results showed that most British swimmers who reached the finals had competed in 200 m events. However, it is necessary to clarify that this could be due to the participation in multiple events of one (or more) high-level swimmer(s). Moreover, the largest proportion of swimmers competing in both categories was reached for those who competed in 200 m events.

The results also indicated that the percentage of females who reached the finals decreased as the event distance increased. That is, women reached the finals mostly in 50, 100 and 200 m events. In this respect, it would be informative in future studies to analyze the age of the first participation as well as the years of experience for both males and females. These results match those of Knechtle et al. [26] relating swimming performance between males and females and confirming that the sex difference decreased as the race distance lengthened in elite swimming. The sex difference in relation to pool swimming and open-water swimming performance depends largely on the parameters analyzed, according to Knechtle et al. [26] (i.e., competition level, swimming stroke, and distance). It may also depend on the psychological interaction between the two sexes concerning the feeling of being better than your competitors [27]. 

Several factors may be important in the link between early and later success in elite competitions. In the present study, apart from the C3 swimmers analyzed, another 5 male and 7 females swimmers managed to enter the finals as juniors but did not compete at WCs as seniors. It is not clear whether these swimmers had quit swimming, were injured or were simply not successful enough to qualify for senior WCs. Similarly, of the 60 swimmers who participated in senior WCs, 49 did not participate in junior WCs. We have analyzed all the data available on British swimmers competing in junior and senior WCs, but we could not determine whether some swimmer development was haphazard, or even random, creating champions by chance whereas others cases were planned, systematic, and developmentally appropriate.

Therefore, several limitations that could provide information from a wider number of factors affecting swimmer’s career should be considered in future studies. The analysis of specific individual training programs of elite British swimmers according to the transition from junior to senior could provide clues concerning the success of these swimmers. Pollock et al. [15], analyzing the distance-specific training load of 18 British elite swimmers, reported data of annual and weekly training load and content compared with swimmers competing in sprint, middle-, and long-distance events. The inclusion of data for the Olympic Games could afford a larger picture of the long-term swimmers’ development in Britain. 

The present research provides information to policymakers about the early success in junior category and subsequent success in senior category of Britain in elite swimming. In this respect, the governing body should explore the critical success factors which will enable Britain to assess how best to manage its future success in an increasingly competitive international environment.

Future investigations into this topic should determine the long-term effects of individual elite swimmers and their specific trajectories related to training programs and training practices of world class athletes in successful countries in swimming, as well as the effects of interactions between different factors which have effects on the sport system of a country.

## 5. Conclusions

The number of swimmers who participated in a junior category before a senior category was quite low and the governing body should explore the critical factors influencing the interaction between early success in junior categories and subsequent success in senior categories in this country. In addition, females who participated in both junior and senior WCs were likely to reach top positions in the senior category.

## Figures and Tables

**Table 1:** Characterization of C3 British swimmers by distance and swim stroke.

Distance	Swim Stroke	N of Participants by Gender	% of Swimmers	Final Reached
50 m (n = 6, 5 W, 1 M)	1	1 W	9.09	2 W (33.3%)
	2	2 W	18.18	
	3	0	0	
	4	3(2 W, 1 M)	27.27	
	5	n/a	n/a	
100 m (n = 6, 5 W, 1 M)	1	3 (2 W/1 M)	27.27	4 (3 W, 1 M, 50%)
	2	3 (2 W/1 M)	27.27	
	3	1 W	9.09	
	4	0	0.00	
	5	n/a	n/a	
200 m (n = 6, 5 W, 1 M)	1	3 (1 W/2 M)	27.27	7 (4 W, 3 M, 77.77%)
	2	3 (1 W/2 M)	27.27	
	3	3 (2 W/1 M)	27.27	
	4	4 (3 W/1 M)	36.36	
	5	3 (1 W/2 M)	27.27	
400 m (n = 6, 5 W, 1 M)	1	2 (1 W/1 M)	18.18	3 M (75%)
	2	n/a	n/a	
	3	n/a	n/a	
	4	n/a	n/a	
	5	2 (1 W/1 M)	18.18	

Style 1: freestyle; style 2: backstroke; style 3: breaststroke; style 4: butterfly: style 5 individual medleys; “W” Number of females; “M” Number of males.

**Table 2:** Coefficients from linear regression model.

Gender	Swim Stroke	Statistics				95% CI for B	
		B	SE	T	Sig.	Lower Limit	Upper Limit
Males	Constant	0.515	0.202	2.553	0.011 *	0.118	0.911
	Category	0.081	0.110	0.734	0.463	−0.136	0.297
	2	−1.095	0.149	−7.371	0.000 ***	−1.387	−0.803
	3	−0.959	0.145	−6.594	0.000 ***	−1.245	−0.673
	4	−1.085	0.166	−6.544	0.000 ***	−1.411	−0.759
	5	−0.291	0.159	−1.832	0.068	−0.604	0.021
Females	Constant	0.170	0.141	1.204	0.229	−0.107	0.447
	Category	0.199	0.076	2.611	0.009 **	0.049	0.350
	2	−0.839	0.103	−8.108	0.000 ***	−1.042	−0.636
	3	−0.802	0.116	−6.890	0.000 ***	−1.031	−0.574
	4	−0.801	0.116	−6.906	0.000 ***	−1.029	−0.573
	5	−0.074	0.134	−0.547	0.584	−0.338	0.191

Statistical significance level: * *p* < 0.05; ** *p* < 0.01; *** *p* < 0.001; Swim stroke 2: backstroke; 3: breaststroke; 4: Butterfly; 5: individual medley.
